# Depressive symptoms may be associated with semantic memory decline in elderly adults

**DOI:** 10.1590/1980-57642021dn15-030006

**Published:** 2021

**Authors:** Mariana Faoro, Amer Cavalheiro Hamdan

**Affiliations:** 1 Psychology Graduate Program, Department of Psychology, Universidade Federal do Paraná – Curitiba, PR, Brazil.

**Keywords:** depression, elderly, memory, semantic memory disorders, neuropsychological tests, cognition, depressão, idoso, memória, transtornos da memória, testes neuropsicológicos, cognição

## Abstract

Depressive symptoms are associated with a decline of episodic memory, but the relationship with semantic memory remains unclear.

**Objective:**

The aim of the study was to analyze the relationship of depressive symptoms with the semantic memory in a community-based sample of elderly adults. The sample comprised two groups, namely, clinical (with depressive symptoms) and control.

**Methods:**

The following instruments were used General Evaluation Questionnaire, Montreal Cognitive Assessment-Basic, Wechsler Abbreviate Scale of Intelligence, Geriatric Depression Scale-30 (GDS-30), Beck Depression Inventory-II, Boston Nomination Test (BNT), vocabulary, verbal fluency test (fruits and animals), and Brief Cognitive Screening Battery.

**Results:**

The results showed a significant difference between groups only in BNT. A significant correlation was observed between the BNT and GDS-30. Participants with symptoms of severe depression performed poorly on BNT when compared with individuals with mild symptoms.

**Conclusion:**

These results support the hypothesis that depressive symptoms in elderly adults can affect semantic memory and may worsen with the severity of symptoms.

## RESUMO

Sintomas depressivos estão associados ao declínio da memória episódica, mas sua relação com a memória semântica permanece obscura.

**Objetivo:**

O objetivo deste estudo foi analisar a relação dos sintomas depressivos à memória semântica em idosos que vivem na comunidade. A amostra foi composta de dois grupos: clínico (com sintomas depressivos) e controle.

**Método:**

Foram utilizados os seguintes instrumentos: Questionário de Avaliação Geral, Montreal Cognitive Assessment-Basic (MoCA-B), Wechsler Abbreviate Scale of Intelligence (WASI), Geriatric Depression Scale-30 (GDS-30), Beck Depression Inventory-II (BDI-II), Teste de Nomeação de Boston (TNB), Vocabulário, Fluência Verbal (Frutas e Animais) e Bateria Breve de Rastreio Cognitivo.

**Resultados:**

Os resultados evidenciaram diferença significativa entre os grupos apenas no BNT. Foi observada uma correlação significativa entre TNB e GDS-30. Os participantes com sintomas de depressão grave tiveram um desempenho pior no BNT, quando comparados aos indivíduos com sintomas leves.

**Conclusão:**

Esses resultados apoiam a hipótese de que os sintomas depressivos em idosos podem afetar a memória semântica e aumentam com a gravidade dos sintomas.

## INTRODUCTION

Depressive symptoms and cognitive impairment are the most common complaints in elderly people. It has a high incidence in the elderly people and can be considered a risk factor for the development of dementia.^1^ ^,^ ^2^ ^,^ ^3^ ^,^ ^4^ ^,^ ^5^ A meta-analysis showed that depression in patients with mild cognitive impairment is 32%, and cognitive deficits were found more often in elderly depressed people than in younger depressed adults.^6^ ^,^ ^7^ In contrast, semantic memory deficits of 12 years before diagnosing Alzheimer’s disease were established.^8^

Depressive symptoms can affect cognition in elderly adults. According to Naismith et al.,^9^ the performance of participants with depression were significantly lower than the control group in executive functions, psychomotor speed, verbal learning, and memory. Some studies have shown that semantic memory is affected by the presence of depressive symptoms.^10^ ^,^ ^11^ ^,^ ^12^ Brunet et al.^10^ conducted a study to verify the relationship between depressive symptoms and semantic memory in elderly people with mild cognitive impairment-amnestic (MCIa) and depression. The results showed that depressive symptoms modulate the presentation of semantic memory deficits in people with MCIa.

In another study, Callahan et al.^11^ evaluated semantic memory impairment for knowledge of natural and artificial objects in patients with MCIa and depression. The results showed that patients with MCIa and depression present inferior performance compared with the other groups, including the group with depression, in which there were no changes in semantic memory. Vogel et al.^12^ evidenced that people with some type of affective disorder may perform poorly in semantic memory tests. However, Elderkin-Thompson et al.^13^ developed a systematic review to trace the neurocognitive profiles of patients with depression. The findings showed that people with depression tend to underperform in all cognitive domains except in semantic memory and executive skills.

The relationship between depression symptoms and semantic memory remains unclear.^14^ ^,^ ^15^ ^,^ ^16^ ^,^ ^17^ ^,^ ^18^ This study has two objectives, namely, first, to analyze the relationship of depressive symptoms with semantic memory in a community-based sample of elderly adults, and second, to verify the predictive capacity of variables on performance in instruments that assess the semantic memory construct. We considered a hypothesis that the severity of depressive symptoms worsens semantic memory impairment in elderly adults.

## METHODS

### Participants

The recruitment was carried out in a community-based sample of 88 adults. The inclusion criteria were as follows: elderly adults above 60 years of age, both sexes, and literate. The exclusion criteria were as follows: patients possibly diagnosed with dementia and having intelligence quotient (IQ) below 70. A total of 13 participants were excluded: 11 with a probable diagnosis of dementia and 2 with IQ below 70. Then, the volunteers were divided into two groups according to the cutoff point established in the performance on two depression scales: Geriatric Depression Scale-30 (GDS-30)^19^ and Beck Depression Inventory-II (BDI-II).^20^ The cutoff points for identifying depressive symptoms are presented in the description of the instruments discussed in the next section. The final samples comprised 42 volunteers in the control group (without depressive symptoms) and 33 in the clinical group (with depressive symptoms). The clinical group was subdivided according to the severity of depressive symptoms (i.e., mild depressive symptoms and severe depressive symptoms) by means of performance assessed by the BDI-II. Table 1 shows the demographic characteristics of the sample divided by groups.

**Table 1.** Characteristics of the clinical group and the control group.

	Clinical group (M±SD)	Control group (M±SD)	t-test/χ^2^	p-value
Age	67.5±5.87	68.8±6.38	-0.904	0.369
Sex (F/M)	(29/04)	(38/03)	0.577*	0.693
Schooling	10.7±4.67	12.4±5.1	− 1.515	0.134
IQ	95.5±14.5	99.8±14	− 1.303	0.197
GDS	15.9±4.7	5.17±3.56	11.26	<0.001
BDI	21.9±8.78	6.64±3.97	10.07	<0.001

M: mean; SD: standard deviation; M: male; F: female; GDS: Geriatric Depression Scale; BDI: Beck Depression Inventory; IQ: intelligence quotient. *Fisher’s exact test.

### Instruments

The following instruments were used to evaluate participants, divided according to the function evaluated.

General Evaluation Questionnaire: This instrument was designed specifically for this research to obtain general information about the participant, including demographic data, previous diagnoses, family history, awareness of general health, and medication use.

Montreal Cognitive Assessment-Basic (MoCA-B): Originally developed by Nasreddine,^21^ this cognitive screening instrument was developed for low-educated population. It includes assessing the same cognitive domains (i.e., executive functions, language, orientation, calculation, abstraction, memory, visual perception, attention, and concentration) as the original MoCA, divided into 10 items that add up to 30 points.

Wechsler Abbreviated Scale of Intelligence (WASI): It was developed by Wechsler^22^ as a brief measure of intelligence and adapted for the Brazilian population by Trentini et al.^23^ The battery is composed of four subtests, which evaluate verbal and cognitive execution skills. This research used two subtests, namely, vocabulary and matrix reasoning, to obtain the total IQ. In addition to general intelligence, vocabulary was used in the evaluation of semantic memory.^22^ ^,^ ^23^ It consists of 42 items that the subject needs to define orally; the score ranges from 0 to 2 in each word according to the accuracy of the answers.

GDS-30: Originally developed by Yesavage et al.,^19^ this test is used for detecting depressive symptoms in the elderly population (above 60 years). It consists of 30 items, for which the participants must answer only yes or no, thinking about how they felt the previous week. According to the Brazilian scale validation research,^24^ 10 was adopted as the cutoff point, that is, the participants who obtained the highest score were present in the clinical group. The GDS-30 is more reliable than its reduced form GDS-15, so the original form was used.^25^

BDI-II: It is an inventory created by Beck et al.^20^to measure the severity of depressive symptoms and adapted to the Brazilian population by Gorenstein et al.^26^ In the present research, the revised version BDI-II^27^ was used. It is a self-report scale with 21 items and a score ranging from 0 to 3; the total score is obtained by summing each item.^28^ According to the application manual, BDI-II score levels are minimum (0–11), mild (12–19), moderate (20–35), and severe (36–63).

Boston Naming Test (BNT): Originally developed by Kaplan et al.,^29^ it is widely used to detect language deficits. The volunteer is presented with 60 pictures, which must be correctly named, with 20 s for each item.^30^ There are two studies of adaptation of the test to Brazilian Portuguese,^30^ ^,^ ^31^ and both agree on the applicability of the test if the educational level of the participant is considered. According to Leite et al.,^32^ the adapted version of BNT has a higher number of hits at all educational levels due to cultural differences and socioeconomic status. Therefore, in the present research, we used the original translated version of the BNT, which showed good internal consistency in adaptation research (α=0.862).^32^ According to adaptation research, the cutoff scores were 34 for the elderly people with at least 3 years of schooling and age of 60–79 years, and for people aged above 80 years, it was 33 points.

Verbal fluency test — animals: it is used to evaluate executive functions and semantic memory. It works by questioning the subject to articulate as many animals as he/she can remember within a minute.^33^ ^,^ ^34^ ^,^ ^35^ The score is given according to the number of words correctly remembered.

Verbal fluency test — fruit: this is one of the MoCA-B subtests used to evaluate executive functions and semantic memory,^36^ and it requires the subject to say as many fruits as he/she can remember in 60 s. Within MoCA-B, the average for a maximum score on this test is 13 words or more.

Brief Cognitive Screening Battery (BCSB): this instrument developed by Nitrini et al.^34^ can evaluate episodic memory, attention, concentration, language, visual perception, construction, and planning. It is a list of 10 figures that the examiner must first name and then memorize. After the three initial presentations of the figure (i.e., 30 s), the participant is instructed to remember as many images as he/she can. Later on (after 5 min), the participant is instructed to recall the figures presented. This score is the long-term memory score (M5 index). Finally, participants are shown a sheet that contains, besides the target figures, others that are intrusive. The task of the participant is to recognize the correct images. The cutoff score, according to Nitrini et al.,^37^ is 7 points on the M5 index.

### Procedure and ethical consideration

All instruments were applied in a single session, lasting around 90 min. The order of application of the instruments were General Evaluation Questionnaire, MoCA-B, WASI, GDS-30, BDI-II, BNT, verbal fluency test — animals, verbal fluency test — fruits, and BCSB.

The research was submitted to the Ethics Committee of the Health Sciences Sector of the Universidade Federal do Paraná (UFPR) and approved by number CAAE: 69654817.6.0000.0102. Through the wide dissemination of research in the media, the volunteers were recruited *via* the UFPR Press Office and evaluated at the Center for Applied Psychology of the university. All participants signed an informed consent form, which had all questions about the research and guaranteed anonymity.

### Statistical analysis

All statistical analyses were performed using the Jamovi software version 0.8.2.3.^38^ For the description of the results, measures of central tendency, mean, and standard deviations were used. The Shapiro-Wilk test was used to assess the normal distribution of variables. The data showed a normal distribution. Subsequently, to compare the means between the control and the clinical groups, the Student’s *t*-test was used. According to the severity of depressive symptoms, the clinical group was subdivided into mild depressive symptoms and severe depressive symptoms. Analysis of variance (ANOVA) was used to compare the symptoms and the severity of the control group. The significance level for rejecting the null hypothesis was p<0.05. To verify the degree of association between the variables, the Pearson correlation coefficient was used. Multiple linear regression (Enter method) was performed to verify the predictive capacity of variables on performance in instruments that assess the semantic memory construct. Finally, to understand the clinical applications of the results, the effect size, Cohen’s *d*, and η² were calculated. According to Cohen,^39^ for Student’s *t*-test, we considered 0.20 (small), 0.50 (medium), and 0.80 (large). For ANOVA, we considered 0.10 (small), 0.25 (medium), and 0.40 (large).

## RESULTS

Table 1 presents the sociodemographic variables of the sample. There are no statistically significant differences regarding the age, education, and IQ of the variables. However, the difference between groups in the depression scales appears significant. The sample was mostly composed of women in both groups.

Significant differences were observed in the performances of the clinical and the control groups only in BNT (Table 2). However, Cohen’s *d* variable shows the average effect size in MoCA-B and BNT and small size in vocabulary and verbal fluency test — fruits.

**Table 2.** Sample performance.

	Clinical group (M±SD)	Control group (M±SD)	t-test	p-value	Cohen’s d
MoCA-B	24.7±3.7	26.07±2.81	−1.821	0.073	−0.426
Vocabulary	45.12±10.3	49.1±9.93	−1.692	0.095	−0.394
BNT	44.48±7.75	48.02±7.45	−1.995	0.05*	−0.467
Animals	15.7±5.07	16.5±4.19	−0.756	0.452	−0.176
Fruits	12.12±2.99	13.14±3.38	−1.368	0.176	−0.318
BCSB	8.21±1.81	8.55±1.92	−0.726	0.471	−0.179

M: mean; SD: standard deviation; MoCA-B: Montreal Cognitive Assessment-Basic; BNT: Boston Naming Test; BCSB: Brief Cognitive Screening Battery. *p<0.05.

In Table 3, the clinical group was subdivided into mild depression (*n*=24) and severe depression (*n*=9) and was compared with the control group. The ANOVA showed significant differences in BNT between the three groups. Post-hoc analysis (Bonferroni test) showed that the difference was significant between the control group and the severe depression group (p=0.04) but not between the mild depression and severe depression groups (p=0.427). The control group and the mild depression group showed no significant difference (p=0.538). The analysis also showed the average effect size in BNT.

**Table 3.** Comparison between the clinical groups and the control group about applied tests.

	Mild depression (M±SD)	Severe depression (M±SD)	Control (M±SD)	F-test	p-value	Ƞ^2^
MoCA-B	24.6±3.63	25.1±3.86	26.1±2,81	1.76	0.179	0.047
Vocabulary	45.5±10.8	42.3±10	49.1±9.93	2.09	0.131	0.054
BNT	45.4±8.04	41.1±6.03	48±7.45	3.39	0.039*	0.086
Animals	15.9±5.17	14.4±4.88	16.5±4.19	0.762	0.470	0.020
Fruits	12.2±3.23	11.3±2.4	13.1±3.38	1.43	0.245	0.038
BCSB	8.33±1.62	7.88±2.23	8.55±1.92	0.456	0.636	0.014

M: mean; SD: standard deviation; MoCA-B: Montreal Cognitive Assessment-Basic; BNT: Boston Naming Test; BCSB: Brief Cognitive Screening Battery. *p<0.05.

The correlations between the tests that evaluate semantic memory and the depression scales are shown in Table 4. In this regard, there is a significant correlation (small) between GDS-30 and BNT (*r*=-0.30; p<0.01).

**Table 4.** Correlations.

	Vocabulary	BNT	Animals	Fruits	BCSB
BNT	0.64***				
Animals	0.51***	0.58***			
Fruits	0.35**	0.48***	0.54***		
BCSB	0.34**	0.51***	0.36**	0.34**	
GDS-30	−0.27*	−0.30**	−0.13	−0.14	−0.04
BDI-II	−0.196	−0.26*	−0.14	−0.05	−0.12

GDS-30: Geriatric Depression Scale-30; BDI-II: Beck Depression Inventory-II; BNT: Boston Naming Test; BCSB: Brief Cognitive Screening Battery. *p<0.05, **p<0.01, and ***p<0.001.

Table 5 presents the multiple linear regression model performed. The age, education, gender, and GDS-30 score of the variables were selected as predictors for the BNT. The model showed *R*-squared=0.61 and adjusted *R*-squared=0.37.

**Table 5.** Multiple linear regression model.

Predictor	Slope	SE	95%CI			
			Lower	Upper	t-test	p-value
Interception	60.089	11.191	37.763	82.4153	5.37	<0.001*
GDS	−0.278	0.114	−0.507	−0.0502	−2.43	0.018
Age	−0.245	0.138	−0.520	0.0298	−1.78	0.080
Schooling	0.629	0.172	0.285	0.9729	3.65	<0.001*
Sex MAL–FEM	3.566	2.606	−1.634	8.7650	1.37	0.176

95%CI: 95% confidence interval; MAL: male; FEM: female; GDS: Geriatric Depression Scale. *p<0.05.

## DISCUSSION

This objective of the study was to analyze the relationship between depressive symptoms and semantic memory in a community-based sample of elderly adults. The results showed a significant difference between the clinical (with depression symptoms) and control groups only in BNT. A significant negative correlation was found between GDS-30 and BNT, indicating that the higher the score on the scale, the lower the BNT score. These results support the initial hypothesis that the severity of depressive symptoms affects the impairment of semantic memory.

The results in the present study corroborate some studies in the literature.^10^ ^,^ ^11^ ^,^ ^12^ On the other hand, these findings contradict some studies.^13^ ^,^ ^40^ The systematic review by Elderkin-Thompson et al.^13^ did not find a decline in semantic memory in patients with depression. Koening et al.^40^ showed a similar result in the verbal fluency test compared with that of people who were never depressed. In the present study, difficulties with verbal fluency were not observed.

An explanation for this discrepancy is related to the characteristics, particularly demographic characteristics, of the clinical samples. Sheline et al.^41^ suggested that age, education, and sex are associated with the GDS-30 score. Another possible explanation is related to the duration of depressive symptoms. Herrmann et al.^42^ showed that participants who have lived with the disorder for a longer time tend to have more significant semantic memory changes than controls, but when compared with patients with depression they perform similarly.

However, how can depressive symptoms affect semantic memory? Semantic memory is a neural network of connections distributed across the sensory, motor, and linguistic areas.^43^ There are three views of how this network would organize: (1) *distributed-only* suggests that it would organize according to the neuroanatomy of related systems; (2) *distributed plus-hub view* defines that there would be a central activation axis formed by a group of amodal neurons that would coordinate the connections and cross information;^44^ and (3) *hub-and-spoke*, which proposed the existence of a radial rather than a distributed model, that is, the amodal center (hub) would be located in the anterior temporal lobe and interact with the rays (spokes) that would be specific regions for the modality.^45^

In other words, the naming task requires the subject to observe the image, activate the sensory, motor, and linguistic areas related to this object, cross the information and correctly identify it, and demand the semantic neural network. Depression implies inhibition of the nervous system; it theorized that semantic connections are also affected by this state, making it more difficult for these patients to access the correct name of the object or, in some cases, increasing the response latency. Neuroimaging studies have found functional abnormalities that affect regulation neurocircuits in patients with depression, involving changes in connectivity between the cortical regions such as the prefrontal cortex (PFC) and subcortical regions amygdala and hippocampus.^46^ When triggered, semantic memory demands the activation of the PFC to evoke information, so, in theory, depression could directly influence this process, and the longer the symptoms last without a proper treatment, the more efficiently the clinical symptoms would be observed.

Cognitive factors, such as recall, semantic representation, and executive functions, in the structure and processing of semantic memory during the aging process are still a topic of debate.^47^ Some studies have shown that executive functions have a “top-down” effect on basic cognitive processes in the presence of depressive symptoms.^48^ ^,^ ^49^ However, there is evidence that depression alone is due to the impairment of semantic memory.

This study had some limitations. It is important to note that there are many variables to be considered in a patient with depression, for example, when depression was diagnosed, medication, and the effect of nonpharmacological treatments. In this study, these variables were not controlled. A cohort study would allow a more detailed understanding of the effects of depressive symptoms on semantic memory.

In summary, this study analyzed the relationship between depressive symptoms and semantic memory. The results showed that deficits in semantic memory increase in proportion to the severity of depressive symptoms. The implications of this study suggest that in cognitive assessments and the presence of depressive symptoms in the elderly people, possible deficits in semantic memory should be considered.
